# Items

**Published:** 1872-12

**Authors:** J. H. Etheridge

**Affiliations:** Chicago


ARTICLE III.—*Items.* Collated by J. H. ETHERIDGE, M.D., Chicago.

"ASTRONOMICAL ETIOLOGY."

Dr. M. L. Knapp, a veteran worker and observer in medical science, writes to the *New York Medical Journal* from Cadereyta, Nueva Leon, Mexico, on the above subject. A *short* review of the article is difficult, because the subject is so vast. His article covers about thirty pages of the above journal, and every line of it is absorbingly interesting. The drift of his argument is to prove a *planetary* cause for the visitations of epidemics, epizootics, blights in vegetation (and consequent famines), etc., etc. The sun, besides being the source of light, heat and electricity, possesses an *attractive* influence over the earth, making the enormous tidal waves of the ocean. The moon also possesses the latter influence, and being nearer to us than the sun, raises higher tidal waves than the larger luminary. This force of gravity is well known to *diminish* as the square of the distance between two sidereal bodies *increases.* Next to the sun, astronomers tell us that the planet Jupiter is by far the most influential element in the planetary system over us. Saturn comes next, in its influence over the earth. These two planets in making their perihelion circuits "must affect the earth and the organized existences on its surface to a considerable extent or degree, by their increased attraction of gravitation and disturbance of its atmosphere, and the natural vital stimulants of all organized life" on the earth. "The same is philosophically true of the approaches of all the other planets, in proportion to their masses and proximity. When all approach coincidently, the combined effect appears by the illustrations which I will offer to be very disastrous to the well being of man, animals, and the crops and the fruits of the earth."

Blights in vegetation invariably precede and accompany epidemics. These coincidences have been observed in all time, but no one has explained them. "What causes them?" has so vexed the thinking world that the unsatisfactory answer has been forced from us, "*God only knows.*" "It is clear to me now," the writer says, "though I may fail to convince others, that the recurrence of the blighting years and meteorological inharmonies that cause famines and epidemics are chargeable to *planetary* influences." In attempting to account for unusual vicissitudes, excessive heat, cold, droughts, and continued rains, observers seem never to have connected them with the perihelia of the larger planets, (either alone or singly,) when there were consequent powerful disturbances of meteorological conditions. Heat and light are natural stimuli, without which the organisms of animal and vegetable life cannot exist. The temperate application of these seems to be the rule favorable to life, "or, in other words, moderate impressions (of light and heat) are favorable to life and health, and extreme impressions injurious." The force of gravity, greatly increased at times by the perihelion of the larger planets, plays an "important part in the universe as heat or light, * * * and I shall soon illustrate that the *pestilential periods are always coincident with the perihelia of the larger superior planets, especially of Jupiter and Saturn.*" Preceding and accompanying epidemics there are, invariably, "malign, cosmical phenomena," and these phenomena are synchronous with the near approach of the larger planets to the earth and sun, which approach Dr. K. believes to be the sole cause of the great disturbance of the earth's atmosphere. Ascertaining the time of the perihelia of Jupiter and Saturn, and reviewing the recorded history of epidemics, reveal a startling coincidence of atmospheric disturbance and epidemics. "The revolutions of Jupiter, the most disturbing element of the system, seem to govern the recurrence of the pestilential periods. His period of revolution is 11 years and 315 days—somewhat less than 12 years—and this interval of time corresponds most remarkably to the interval of recurrence of the pestilential periods."

Since Jupiter's perihelion in the year 1572, the earth has been visited, every twelve years—dates tallying exactly with recurrences of the near approach of this planet—"by aggravated pestilence the world over." The last visitation was in 1868, and everybody recalls the cholera of that year. Saturn's return is every 29 years and about 167 days, and each perihelion of this planet is likewise marked by pestilential visitations. Every fifth perihelion of Jupiter occurs in the same year with that of Saturn, and these periods "I find, without exception, to be *remarkably aggravated pestilential periods*, extending generally through a whole revolution of Jupiter, or even to 15 years or more duration. These periods will constitute my examples. They are bold and unmistakably pronounced. They occur in cycles of about 59 years—59 years less 16 days. * * * Five of these commensurate periods have occurred within the last 300 years." (The writer does not go farther back than three centuries, feeling that that period is long enough to test his theory.) The first one of these five periods was in 1620. During this cycle there were "great disturbances in the physical world, and severe epidemics. * * * The Virginia Colonies were nearly depopulated. The North American Indians nearly all died. The Pilgrims found their bones bleaching above ground in 1620." The next period of the perihelia of Saturn and Jupiter was in the year 1679. "In 1677 small pox raged in Massachusetts with the mortality of the plague. In 1678 fevers and anginas were epidemic in Europe, and authors relate that four millions of people perished that year in Africa of plague." At the next period of perihelia of these two planets, in the year 1738, there were well marked pestilential periods. Passing on over another period of 59 years we come to the frightfully pestilential period of 1797. During this period of perihelia Noah Webster wrote his exhaustive "History of Pestilence,"—the work being prompted by the epidemics raging at that time. This was in the day of Dr. Rush, and any one familiar with his writings recalls this period. During the summer of 1798 the yellow fever was epidemic in every Atlantic seaport to Portland, Maine. In April, 1797, Saturn's proximity to the earth was the greatest, and that of Jupiter occurred in August of the same year. In the August of the *next* year, 1798, Uranus also made his perihelion, and thus three planets were in close proximity to our poor earth, and the combined influence of them all was synchronous with a remarkably pestilential period. The last perihelion epoch the writer calls our attention to was in 1856. "The five periods of Jupiter [previous to 1856, while Saturn was approaching. E.] were 1856, 1845, 1833, 1821, and 1809. Most of my readers will recollect the yellow fever epidemics of 1853 in New Orleans, 1855 in Norfolk, and 1856 in New Orleans again with greater severity; and the ship fever epidemics of the Irish famine years from 1845 to 1849, when they culminated in cholera; as well as the general pestilence, all over the world, of this period, extending through to 1856."

In reviewing this article I have enumerated only the near approach of these two planets conjointly, omitting, for brevity, the frequent perihelia of Jupiter, which the author has carefully noticed in his essay, for nearly three centuries, in every instance. Rarely have there been more than two planets near the earth at the same time, and according to Dr. Knapp's theory the more planets in their perihelia simultaneously the more pestilential the period on the earth. Such a time occurred in 1798, when Saturn, Jupiter and Uranus were in their perihelia. Of course such events are rare, as the farther from the sun we go the seldomer the return of the planets. Leaning on this theory we certainly have much to expect in the line of epidemics in the future. Taking the approaches of Jupiter alone—leaving Saturn out of calculation—we shall find every twelfth year—the year of his return—characterized by epidemic visitations to an astronomical certainty. His last perihelion was in 1868; his succeeding ones, in round numbers, will be in 1880, 1892, 1904, and so on. Hence our next epidemic period may be looked for in 1880. But if Dr. K. be correct in his propositions there is a terribly "sable period not far distant in the future,"—in fact, commencing in 1880 and extending over a period of five or eight years. Jupiter's perihelion occurs in 1880, and before he ceases exerting his powerful influence over us, he and Saturn will form a "conjunction," Uranus and Neptune will make their commensurate perihelia in 1882, and Saturn will make his perihelion passage in 1885. "Lively times in physic—lively times for doctors and undertakers also—may be looked for by those who believe in the certainties of astronomy, all the way from now to then, for Uranus will not complete his perihelion circuit until the going out of the 19th century, and Neptune not until 1923; so that malign influences may be looked for under every recurring perihelion approach of Jupiter, during the cycle in which we are now sailing, viz.: 1880, 1892, 1904, and 1916."

RESEARCHES ON THE MOVEMENTS OF THE UTERUS.

In the last part of Stricker's *Medical Jahrbucher*, are recorded some experiments by D. L. Oser and W. Schlesinger, performed in investigating the movements of the uterus. These movements being unknown, and those of the intestines, ureters and other organs being well known, the experiments commenced work in a new field. Curarised rabbits were chiefly used.

The effects of (1) arrested respiration—(2) aortal compression and rapid abstraction of blood—(3) sections of the cord.

(1) Krause, Meyer and Basch have all shown that asphyxia incites intestinal movements. No one has shown that the uterus is similarly influenced—but Oser and Schlesinger show, that contractions of this organ, commencing from the tubae and cervix and soon extending over the whole organ, supervene in from ten to thirty seconds after suspension of respiration. The organ becomes pale and rigid, and moves downwards and towards the mesial line. The contractions increase in vigor with the continuation of the asphyxia for some moments.

(2) Aortal compression, rapid abstraction of blood, and compression suddenly applied to the carotids and vertebrals, produced general contractions of the uterus in from 80 to 120 seconds.

(3) Sections of the cord showed that uterine contractions occurred in about 100 seconds whether asphyxia or aortal compression were resorted to, and that *no* uterine contractions followed when there was general loss of blood or compression of the cerebral arteries. The brain does not send any stimulus to the uterus along the vagi, since section of them produced no influence on the uterine movements, whether the cord were sectioned or not. From their experiments they think that the uterus nerve centre lies in the medulla, and not in the cord lower down.

ACUTE ARTICULAR RHEUMATISM TREATED WITH IMMOVABLE APPARATUS.

Prof. Luigi Concato, of Bologna, Italy, advocates this treatment for inflammatory rheumatism, claiming for it a marked superiority over any other treatment, which certainly seems very reasonable. He says: "It fixes the articular extremities immobile, and thus prevents the torturing pain of motion. It compresses the articulation uniformly and continuously, and thus antagonizes the distension of the soft parts and checks the disposition to exudation. It favors by its direct pressure force, the reabsorption of exudation, and consequently abbreviates the duration of the disease. In this way it prevents complications of pericarditis, endocarditis, etc." It produces its beneficent effects at once, giving relief from the constant fear of moving which the patient always has, and in a great degree this immobility relieves actual pain.—(*The Clinic.*)

NEW METHOD OF DETECTING SMALL POX.

An Italian physician can detect the presence of variola before the appearance of the eruption by an itching on his forehead and chin! He has verified the statement by a number of observations, and his assistants also confirm it by their own experience.—(*Pacific Medical and Surgical Journal.*)

APPLICATION FOR CHILBLAINS.

Two parts oxide; one part tannic acid; ten parts glycerine; eight parts balsam Peru; four parts camphor; to be applied night and morning.—(*Union Med.*, Oct. 15.)

POMADE IN LOSS OF HAIR.

M. Bouchut recommends the following, to be rubbed in night and morning, when the hair falls off after delivery or serious illness, giving at the same time, internally, iron and quinine, and in some cases the arseniate of soda: Ten parts extract henbane; five parts tincture of iodine; thirty parts beef marrow; scenting with bergamot.—(*Ib.*)

ORCHITIS TREATED WITH ABSOLUTE REST.

Dr. Brambilla, of Lodi, Italy, publishes in the *Lombard Medical Gazette*, an account of twenty-two cases of orchitis treated by this means—twelve of these were gonorrhœal, six traumatic, and four idiopathic. In all the cure was complete. Medium time of treatment was five days, "although on admission the affection had lasted from two to five or six days, and the testis had acquired from two to five times its normal size. None were dismissed until the organ had reacquired its normal size." "By absolute rest is meant that the patient should lie continuously on his back, with a small cushion between the thighs, in order to support the testis, and that he must never get off the bed even for the purpose of attention to his natural wants." The Dr. thinks orchitis can be more rapidly and more effectually cured in this way than by any of the ordinary means. —(*London Medical Times and Gazette.*)

### OINTMENT FOR PILES.

M. F. Guym, of the Neeker Hospital, Paris, prescribes, in painful hæmorrhoids, an ointment compounded of one part extract belladonna, two parts extract rhatany, and fifteen parts lard.—(*The Doctor*, August, 1872.)

### LIME FOR POISONING BY PLANTS AND INSECTS.

A standing antidote for poisoning by oak, ivy, etc., is to take a handful of quicklime, dissolve in water, let it stand half an hour, then paint the poisoned parts with it. Three or four applications will never fail to cure the most aggravated cases. Poison from bees, hornets, spider bites, etc., is instantly arrested by the application of equal parts of common salt and bicarbonate of soda, well rubbed in on the place bitten or stung.—(*Boston Jour. Chemistry.*)

### INGROWING TOE NAIL.

About twenty years ago I applied a bit of compressed sponge to afford temporary relief, and was delighted to find that it effected a radical cure. I make the sponge as solid as leather, by wetting and then winding a string tightly around it and drying it thoroughly. Of this I cut a small pyramidal piece, less than a grain of rice. This I insert beneath the nail and secure by strips of adhesive plaster, applied longitudinally, to avoid compression. The sponge soon becomes moist and swollen, keeping the nail from the irritated flesh. Any granulations should be previously destroyed with strong nitric acid. I have adopted this plan upon many occasions and have never found it to fail.—(Benj. Blower, *British Med. Jour. The Clinic.*)

### POSITIVE SIGN OF DEATH.

Dr. Hugo Magnus, of Breslau, suggests the following " simple physiological and conclusive 'test': All that one has to do, therefore, is to *tie a string firmly around the finger of the supposed corpse.* If there is the least spark of life left, that is, if the blood circulates at all, the whole finger from the string to the tip will gradually turn a bluish red, from the engorgement of the veins. Nothing else, no *post mortem* infiltration, can be mistaken for this appearance."—(*Philadelphia Medical and Surgical Reporter.*)

NEW METHOD OF FORCED DILATATION FOR THE TREATMENT OF ANAL FISSURE.

The patient being chloroformed, a small bivalve speculum uteri is introduced into the rectum, which when closed is only three centimetres in diameter, but when completely opened becomes eight centimetres. This is easily introduced when closed, and, being opened, it can only be withdrawn by use of considerable force. The resistance being overcome, the parts are distended, and a cure is at once effected.—(Dr. Alf. Liegard, *La Tribune Medicale*, April, 1872. *Am. Jour. Med. Sciences. Med. Archives.*)

